# Theoretical and *in silico* Analyses Reveal MYC as a Dynamic Network Biomarker in Colon and Rectal Cancer

**DOI:** 10.3389/fgene.2020.555540

**Published:** 2020-10-20

**Authors:** Yanqiu Tong, Yang Song, Chuanhui Xia, Shixiong Deng

**Affiliations:** ^1^Department of Broadcasting and TV, Chongqing Jiaotong University, Chongqing, China; ^2^Laboratory of Forensic Medicine and Biomedical Informatics, Chongqing Medical University, Chongqing, China; ^3^Department of Medical Informatics, Chongqing Medical University, Chongqing, China; ^4^School of Materials Science and Engineering, Chongqing Jiaotong University, Chongqing, China

**Keywords:** dynamic network biomarker, colon and rectal cancer, time-course gene expression data, tumor suppressor genes, immune infiltrates

## Abstract

In this article, we make a theoretical and *in silico* study for uncovering and evaluating biomarkers in colon and rectal cancer (CRC) by the dynamic network biomarker (DNB) theory. We propose a strategy to employ the theoretical concept of UICC TNM classification in CRC. To reveal the critical transition of CRC, the DNB algorithm was implemented to analyze the genome-wide dynamic network through temporal gene expression data. The relationship between gene sets and clinical features was evaluated by weighted gene co-expression network analysis. The results show that MYC was significantly associated with tumor amplification, tumor immune cells, and survival times. The candidate tumor suppressor genes were ZBTB16, MAL, LIFR, and SLIT2. Protein–protein interaction (PPI) analysis shows that these candidate tumor suppressor genes were significant in immune cells. Data from the Human Protein Atlas showed that a high expression of these candidate tumor suppressor genes was associated with favorable prognosis in TNM stages I–IV. In conclusion, this work provides significant and novel information regarding the TNM stage, cause, and consequences of elevated MYC expression in CRC. MYC expression levels had significant negative correlations with tumor suppressor genes and immune cells.

## Introduction

Colon and rectal cancer (CRC) is one of the most frequently diagnosed tumor in terms of incidence, and its incidence among young people has increased in recent years (Brenner et al., 2019; Dang et al., 2020). Over 1.8 million of new CRC cases and 881,000 deaths were estimated to occur in 2018 (Bray et al., 2018). Despite the advance technology in CRC screening and treatment strategies such as molecular biomarkers and immune therapy, poor prognosis remains significant (Wang et al., 2019). Lots of complex factors cause the occurrence and the progression of CRC, such as age, family history, gender, region, and tumor stage (Center et al., 2009; Peng et al., 2018; Tong et al., 2018).

Colon and rectal cancer is a genetically heterogeneous disease in which several key biomarkers in different tumor stages could play an important function in tumor initiation, growth, and progression (Ciardiello et al., 2019). It is reported that 40% of CRC patients have disease metastases (Tsikitis et al., 2014); 80% of them metastasize to the liver, and the remaining 20% metastasize to other visceral organs (Manfredi et al., 2006), so cancers diagnosed in early stage are important for the prognosis of CRC, but patients diagnosed at later stages are the majority (Dupaul-Chicoine et al., 2015).

Immune infiltrates in cancer play the key function in different tumor stages from tumorigenesis to tumor development (Balkwill and Coussens, 2004; Castell and Larsson, 2015). With a better understanding of tumor cells and the immune system, a new tumor treatment method, immunotherapy, brings hope to tumor patients (Ciardiello et al., 2019).

Recently, high-throughput omics, molecular biology, and computer technology, have provided us a computable perspective, revealing the molecular characteristics of occurrence, development, and prognosis in CRC. Theoretical and *in silico* studies have been predominantly applied for providing novel insights and strategies for cancer biomarker prediction and treatment. Traditionally, molecular biomarkers were identified as tumor samples from normal samples in clinical practices. There are some limitations in molecular biomarker screening (Wen et al., 2014; Xiaoping et al., 2016). To overcome these weaknesses, a network-based algorithm for identifying biomarkers attracts much interest and shows good performance (Liu et al., 2014). However, molecular markers and network-based markers are static analysis methods that distinguish biomarkers from normal samples and tumor samples (Liu et al., 2014). These methods cannot satisfy for predicting pre-disease or the inflection point of tumor stage transition, so there is a need for the prediction method with the function of dynamic prediction characteristics in pre-disease development and critical time–life cycle evolution.

In this study, we adopt the dynamic network biomarker (DNB) algorithm to identify the DNBs. The foundation of DNB algorithm was based on nonlinear dynamical theory and complex network theory (Wong et al., 2017). Biomarkers play a critical role in living organisms, which can be used to indicate and evaluate the biological process or drug targets for diagnosis or treatment. The math model for detecting DNBs can be summarized as the following conditions (Chen et al., 2015): from the system perspective, DNB is a system that nears the tipping point, which exists as a dominant group and is called DNB. The DNB model should satisfy the following four critical conditions (Liu et al., 2014, 2018; Zhang et al., 2019):

1. Deviation for each DNB members is becoming highly fluctuating (SD_in_: standard deviation for input).
2. Correlations among all members of DNB are increasing drastically (PCC_in_: Pearson correlation coefficients in absolute values for input).
3. Correlations between any DNB members and non-DNB members rapidly decrease (PCC_out_: Pearson correlation coefficient in absolute values for output).
4. There are no drastic deviations or correlations among all non-DNB members in the system.

The input standard deviation (SD_in_) can be defined as:

$${\text{SD}}_{i\,n}\,\left(x_{d}\right)=\left|x_{d}-\bar{x}\right|$$

where *x*_*d*_ is the expression of gene *x* in the new sample *d*, and $\bar{x}$ is the average expression of gene *x* in the *n* reference samples.

Given *n* reference samples, the Pearson correlation coefficient (PCC) between gene *x* and gene *y* in the reference sample data can be calculated as:

$$\text{PCC}\,\left(x,y\right)=\frac{\sum_{i=1}^{n}\left(x_{i}-\bar{x}\right)\,\left(y_{i}-\bar{y}\right)}{\sqrt{\sum_{i=1}^{n}{\left(x_{i}-\bar{x}\right)}^{2}\,\sum_{i=1}^{n}{\left(y_{i}-\bar{y}\right)}^{2}}}$$

where *x*_*i*_ and *y*_*i*_ are the expression values for gene *x* and *y* for the *i*th sample in the reference samples, respectively. At the same time, $\bar{x}$ and $\bar{y}$ are the average gene expression values of genes *x* and *y* in the reference samples, respectively.

In order to detect a reliable and clear signal of the pre-disease state, the composite index (CI) was used to evaluate the DNB score as follows:

$$\text{CI}={\text{SD}}_{i\,n}\,\frac{{\text{PCC}}_{i\,n}}{{\text{PCC}}_{o\,u\,t}}$$

## Materials and Methods

### Samples

The GSE21510 microarray dataset including gene expression profiles was acquired from the Gene Expression Omnibus (GEO) database. The GSE21510 dataset was employed for identifying a novel biomarker or a target of treatment for CRC using laser microdissection and oligonucleotide microarray analysis (Tsukamoto et al., 2011). The validation dataset was obtained from The Cancer Genome Atlas (TCGA) database. In these datasets, the samples were divided by the TNM staging system associated with CRC (Table 1).

**TABLE 1:** Samples for TNM staging system associated with colon and rectal cancer.

**Dataset type**	**Dataset**	**Stage**	**Patient samples**
Training	GSE21510	Stage I	15
		Stage II	46
		Stage III	39
		Stage IV	23
Validation	The Cancer Genome Atlas colon and rectal cancer gene expression profile	Stage I	6
		Stage II	36
		Stage III	34
		Stage IV	59

### Analysis for Molecular Biomarkers in CRC

In order to search the critical genes in CRC, we utilized limma R package for screening differentially expressed genes (DEGs) between tumor and normal samples divided into different tumor stages. Two of the cutoff criteria of screening were false discovery rate < 0.05 and log_2_-fold change (| log_2_FC|) > 2.0.

Furthermore, STRING online database was used for constructing a protein–protein interaction network to investigate the relationships or connections among the critical genes (Szklarczyk et al., 2019). Because the PPI network was composed of nodes, edges, and degrees, analyzing the topology of the PPI network can help us identify hub genes and key communities. In our research, the PPI networks were analyzed by Cytoscape software with cytoHubba plugin (Shannon, 2003; Chin et al., 2014).

### Network-Based Analysis for Screening Biomarkers in CRC

In this article, the TCGA Colon and Rectal Cancer gene expression profiles were used for evaluating the relationship between DNBs and different tumor stages by combining clinical information in CRC (Zhang and Horvath, 2005). The weighted gene correlated network analysis (WGCNA) method can facilitate the construction of free-scale gene expression networks, and the “WCCNA” R package was used to calculate a standard scale-free network (Langfelder and Horvath, 2008).

### Validate DNBs by Multi-Dataset

To validate the DNBs calculated in GSE21510 and TCGA Colon and Rectal Cancer gene expression profiles, public databases were used as cross-validation. All the databases in this article are listed in Table 2.

**TABLE 2:** Validation databases for colon and rectal cancer.

**Dataset name**	**Data link**	**Reference/s**
GEPIA	http://gepia.cancer-pku.cn/	(Tang et al., 2017)
Human protein Atlas	https://www.proteinatlas.org/	(Uhlén et al., 2015; Thul et al., 2017; Uhlen et al., 2017)
cBioProtal	https://www.cbioportal.org/	(Cerami et al., 2012; Gao et al., 2013)
TIMER	http://cistrome.dfci.harvard.edu/TIMER/	(Li, 2016, 2017)
Ualcan	http://ualcan.path.uab.edu/	(Chandrashekar et al., 2017)
Oncomine	https://www.oncomine.org/	(Selamat et al., 2012)

## Results

### The Identified DNBs for Critical Stage in CRC

**FIGURE 1:**
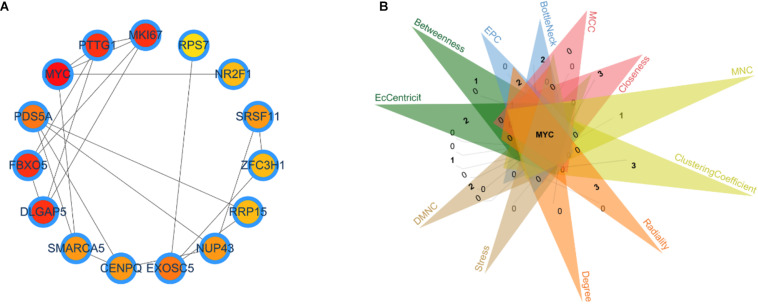
Identification of the critical transition state in colon and rectal cancer. **(A)** Key dynamic network biomarkers (DNBs) screened by DNB algorithms and visualized by protein–protein interaction analysis. **(B)** Venn diagram for 12 types of topological algorithms.

Identifying the different critical transition state in CRC from stage I to IV is crucial for clarifying the molecular mechanism that regulates tumor development. In this article, the DNB algorithm was implemented to search the DNBs. The DNB scores at four stage time points are shown in Table 3. The DNB score increased sharply from stage III and reached the peak at stage IV, so stage III is designated as a critical transition state, which corresponds to the development of a specific stage in CRC. Ultimately, the 98 DNBs from stage I to stage IV were divided into four sampling time points in Figure 1. Then, PPI analysis was constructed by Cytoscape with cytoHubba plug-in. CytoHubba can provide 12 topological analysis methods including betweenness, bottleneck, closeness, clustering coefficient, degree, DMNC, EcCentricity, EPC, MCC, MNC, radiality, and stress based on shortest paths (Chin et al., 2014). Lastly, the Venn diagram analysis showed that the DNB was MYC.

**TABLE 3:** Dynamic network biomarker scores in different tumor stages in colon and rectal cancer.

**TNM stage**	**Score**	**Genes**
Stage I	0.42378583	98
Stage II	0.14771798	98
Stage III	0.09225563	98
Stage IV	0.647891324	98

### Molecular Biomarker Analysis for Different Tumor Stages in CRC

**FIGURE 2:**
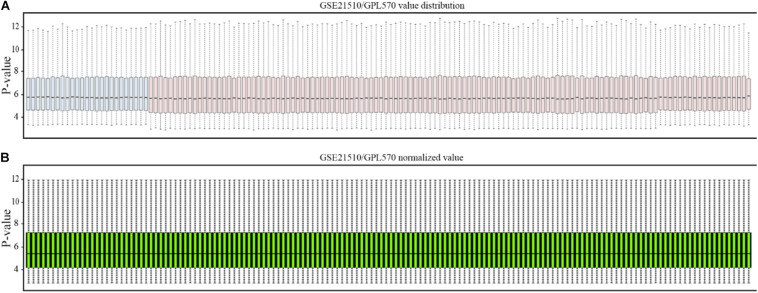
Normalization of gene profiling data. **(A)** Distribution of GSE21510 gene profiling data. **(B)** Value-normalized distribution of GSE21510 gene profiling data.

The affymetrix gene expression profiles (Figure 2A) were analyzed by online GEO2R (https://www.ncbi.nlm.nih.gov/geo/geo2r/) tool, and the normalized results are shown in Figure 2B.

**FIGURE 3:**
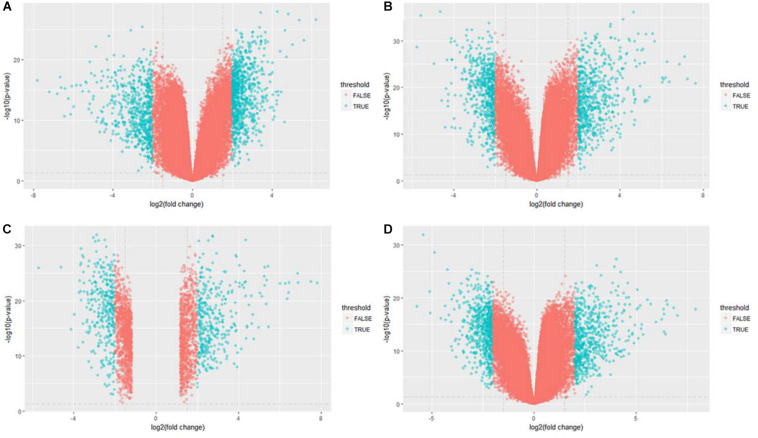
Volcano plot of differentially expressed genes in GSE21510 gene profiling data. **(A)** Stage I. **(B)** Stage II. **(C)** Stage III. **(D)** Stage IV.

After data normalization and removal of batch effects, a total of 5,343 DEGs were identified, of which 2,738 were up-regulated genes and 2,605 were down-regulated genes (Figure 3). The detailed results of the differential expression are shown in Table 4.

**TABLE 4:** Up-regulated and down-regulated genes in stages I, II, III, and IV associated with colon and rectal cancer in GSE21510 dataset.

**Stage**	**Up-regulated**	**Down-regulated**
Stage I	807	640
Stage II	634	607
Stage III	674	715
Stage IV	623	643

### Weighted Gene Correlated Network Analysis for Different Tumor Stages in CRC

**FIGURE 4:**
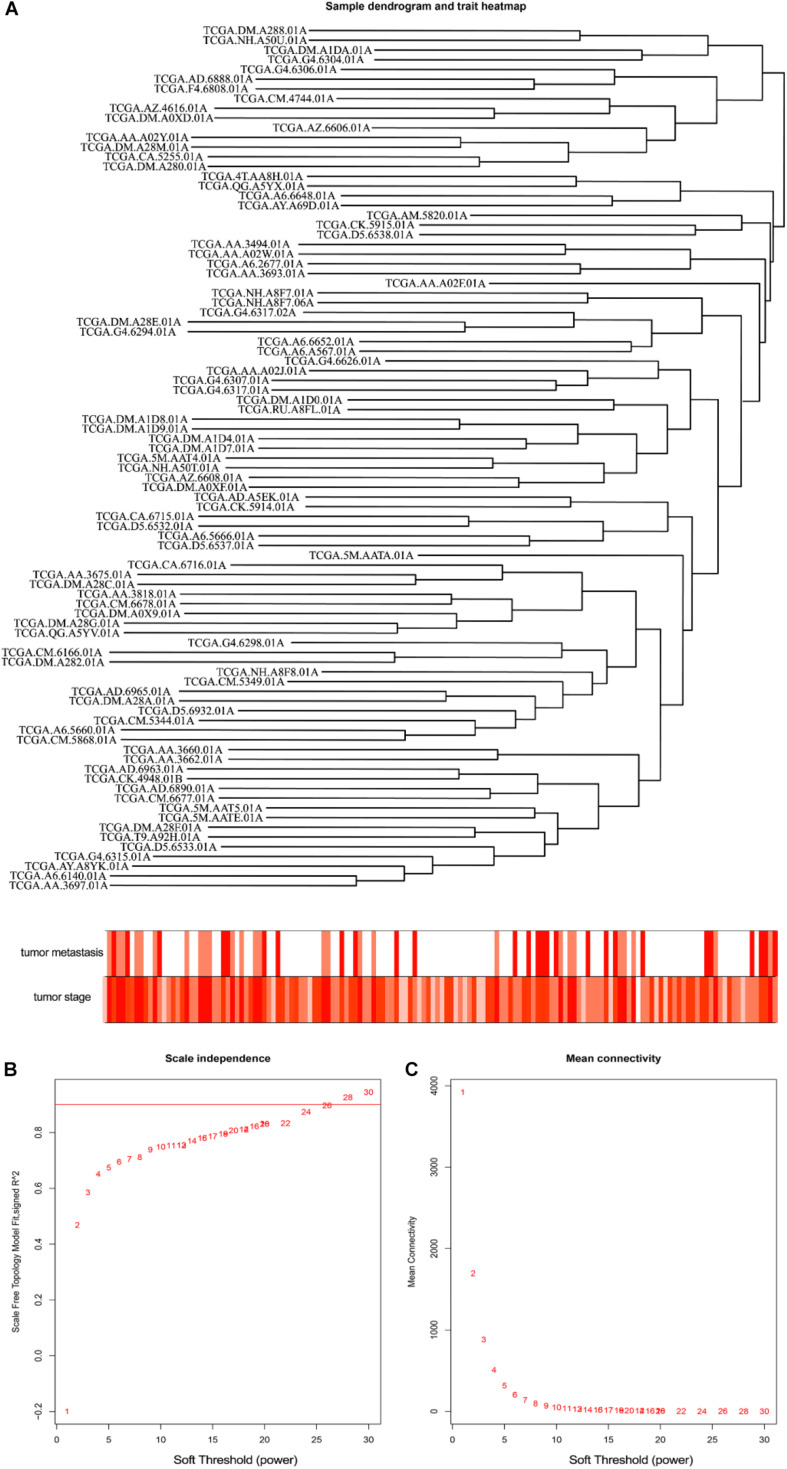
Clustering of colorectal adenocarcinoma (COAD) for soft-thresholding power. **(A)** Clustering of COAD samples. **(B)** Calculating the scale-free fit index of soft-thresholding power. **(C)** Calculating the mean connectivity of soft-thresholding power.

**FIGURE 5:**
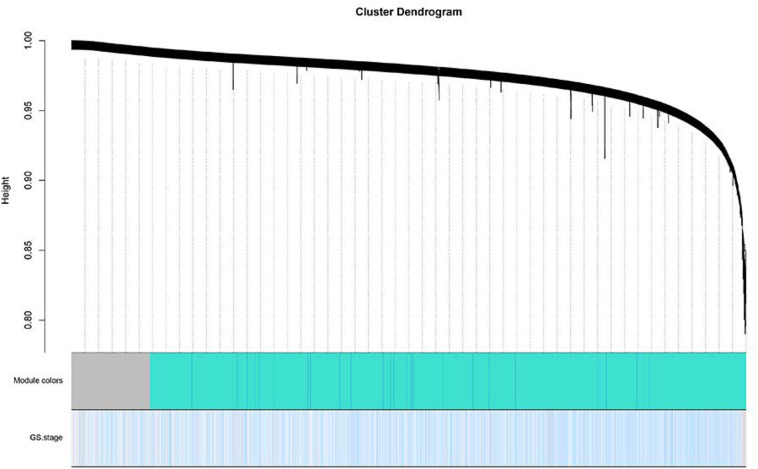
Dendrogram of all differentially expressed genes clustered based on a dissimilarity measure. Dynamic tree cutting was applied to identify modules by dividing the dendrogram at significant branch points. Modules associated with tumor stage are displayed with different colors in the horizontal bar.

**FIGURE 6:**
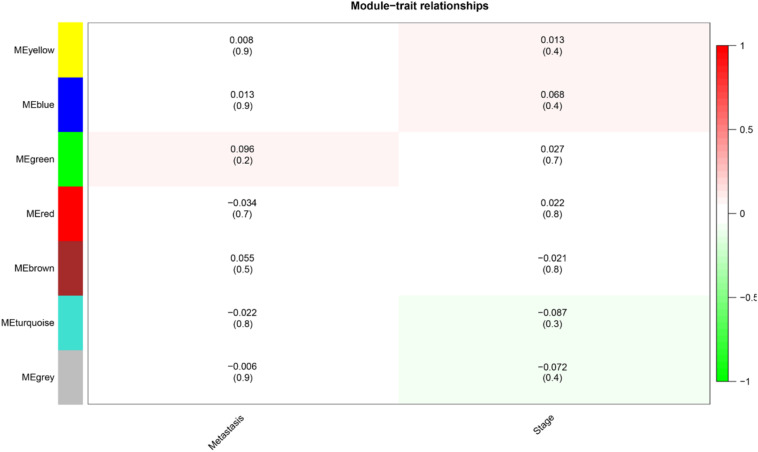
Heat map of the correlation between module eigengenes and clinical subtypes (metastasis and stage) in colorectal adenocarcinoma. Each column showed the corresponding correlation and *P*-value.

The level 3 RNAseqV2 data of colorectal adenocarcinoma (COAD) were downloaded from the TCGA database. The gene expression profiles were generated using the Illumina HiSeq 2000 sequencing platforms. There were 475 samples in total, including 340 tumor and 135 normal samples; these samples were classified into four different stages (Table 5). In order to explore the association between gene sets and clinical features, WGCNA was used to construct free-scale gene co-expression networks (Tang et al., 2018). The samples of COAD were clustered using average linkage method and Pearson’s correlation method (Figure 4A). In this study, the power of β = 6 (scale free *R*^2^ = 0.95) was selected as the soft-thresholding criteria to construct a free-scale network (Figure 4B). A total of seven module eigengenes were identified based on the average linkage hierarchical clustering (Figure 5). The yellow module was found to have the highest correlation with tumor stage (Figure 6), so the yellow module was selected as a clinically significant module for identifying hub genes.

**TABLE 5:** Gene expression profiles in different stages for The Cancer Genome Atlas colon and rectal cancer gene expression profile (the detailed clinical information of colon adenocarcinoma is in Supplementary Table S1).

**Stage**	**TCGA samples**	**Tumor/normal**
Stage I	78	72/6
Stage II	189	153/36
Stage III	135	100/35
Stage IV	73	15/58

### PPI Network and Module Analysis for MYC

**FIGURE 7:**
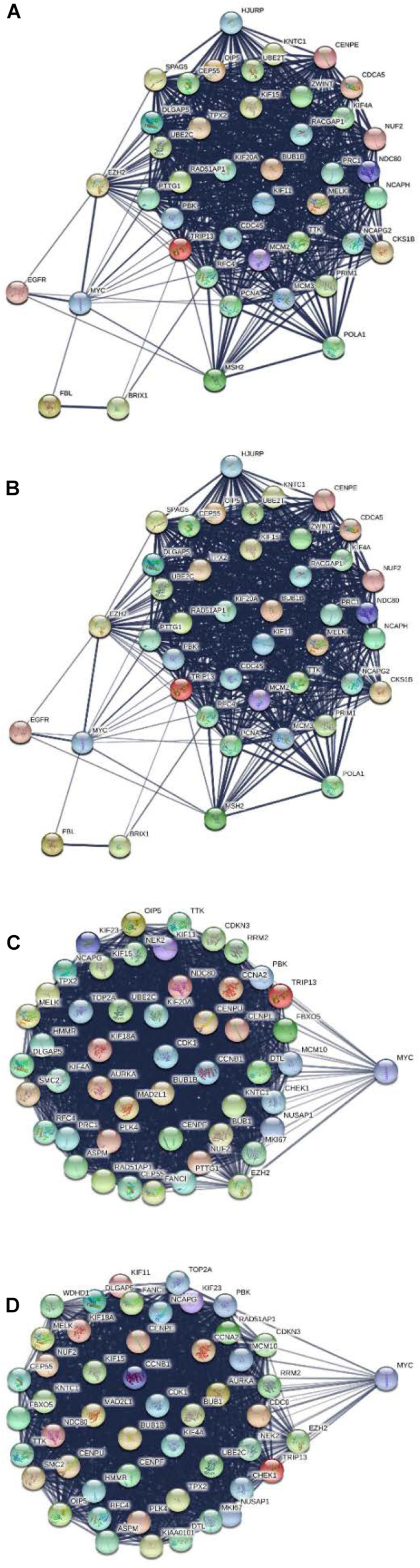
Protein–protein interaction network for MYC in different stages. **(A)** MYC showed significance in stage I. **(B)** MYC showed significance in stage II. **(C)** MYC showed significance in stage III. **(D)** MYC showed significance in stage IV.

All DGEs, yellow module, and DNBs were uploaded to the STRING database for constructing the PPI network. Then, all the nodes and edges were divided by ClusterONE and analyzed by Cytoscape with cytoHubba. The results show that MYC can serve as a dynamic biomarker for CRC (Figure 7).

### Validation of the Dynamic Network Biomarker Gene in TCGA Database Pan-Cancer Analysis for MYC

**FIGURE 8:**
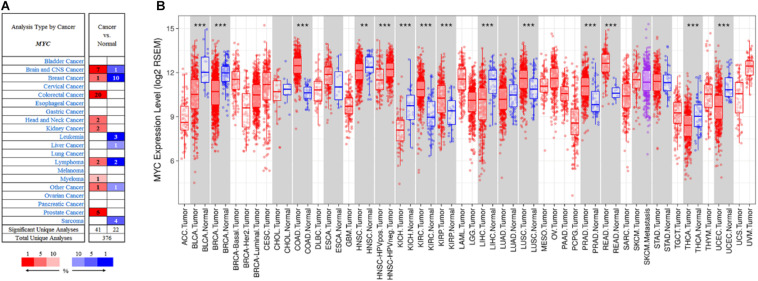
Pan-cancer analysis for MYC. **(A)** MYC expression in Oncomine database. **(B)** MYC expression in TIMER database. The cell color in **(A)** is determined by the best gene rank percentile for the analyses within the cell. Red: tumor. Blue: normal. The box plot colors in **(B)** indicate tumor and normal expression. Red: tumor. Blue: normal. *P*-value significance codes: 0 ≤ *** < 0.001 ≤ ** < 0.01 ≤ * < 0.05 ≤. < 0.1).

The expression of MYC level in different types of cancers was conducted by the Oncomine database (Rhodes et al., 2007). The Oncomine database analysis revealed that MYC mRNA expression of CRC had increased in 20 datasets compared to the normal tissues (Figure 8A). In addition, MYC mRNA expression was lower in breast cancer, head and neck cancer, kidney cancer, lymphoma, and myeloma cancer. Higher expression was observed in brain and CNS cancer, colorectal cancer, and prostate cancer. Pan-cancer analysis showed that MYC expression was significantly higher in colon adenocarcinoma and rectum adenocarcinoma compared with adjacent normal tissues (Figure 8B).

### MYC Expression and Prognosis in CRC

MYC expression was evaluated by the PrognoScan and was notably found to significantly impact prognosis in CRC. Four cohorts (GSE12945, GSE17536, GSE14333, and GSE17537), including different tumor stages of CRC, showed that high MYC expression was associated with poor prognosis (Table 6).

**TABLE 6:** Survival analysis of MYC mRNA in colon and rectal cancer patients (the PrognoScan).

**Dataset**	**Endpoint**	**Number**	**ln(HR_high_/HR_low_)**	**Cox *P*-value**	**ln(HR)**	**HR (95% CI_low_–CI_upp_)**
GSE12945-HG-U133_Plus_2	Disease-free survival	51	15.43	0.875782	0.09	1.09 (0.36–3.35)
	Overall survival	62	−15.38	0.647096	−0.16	0.86 (0.44–1.67)
GSE17536-HG-U133_Plus_2	Disease-free survival	145	−0.65	0.601886	−0.15	0.86 (0.50–1.50)
	Disease-free survival	145	−1.11	0.01759	−3.58	0.03 (0.00–0.54)
	Disease-specific survival	177	−0.97	0.115903	−0.36	0.70 (0.45–1.09)
	Overall survival	177	−0.73	0.216913	−0.24	0.78 (0.53–1.15)
	Overall survival	177	−0.64	0.021105	−2.34	0.10 (0.01–0.70)
	Disease-specific survival	177	−0.69	0.020065	−2.8	0.06 (0.01–0.64)
GSE14333-HG-U133_Plus_2	Disease-free survival	226	−0.44	0.272903	−0.23	0.79 (0.52–1.20)
	Disease-free survival	226	−0.67	0.52951	−0.1	0.90 (0.66–1.24)
GSE17537-HG-U133_Plus_2	Disease-free survival	55	−1.23	0.010481	−5.84	0.00 (0.00–0.25)
	Disease-specific survival	49	−1.81	0.043557	−5.94	0.00 (0.00–0.84)
	Overall survival	55	−0.65	0.823679	−0.06	0.94 (0.56–1.59)
	Overall survival	55	−2.11	0.001411	−7.34	0.00 (0.00–0.06)
	Disease-free survival	55	0.88	0.681468	0.13	1.14 (0.62–2.09)
	Disease-specific survival	49	−0.68	0.96835	−0.02	0.99 (0.47–2.07)

**FIGURE 9:**
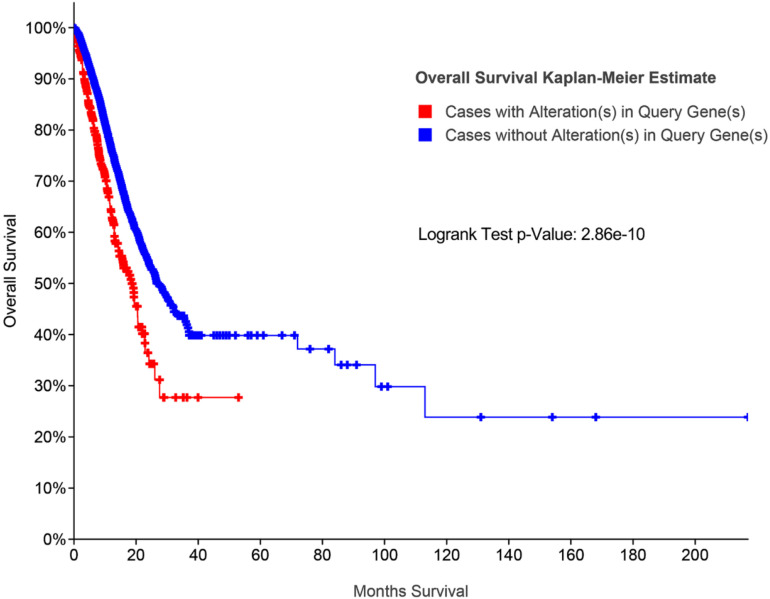
Survival analysis for MYC by cBioPortal database.

Additionally, survival analysis shows that high MYC alteration was associated with poor prognosis in CRC (Figure 9).

**FIGURE 10:**
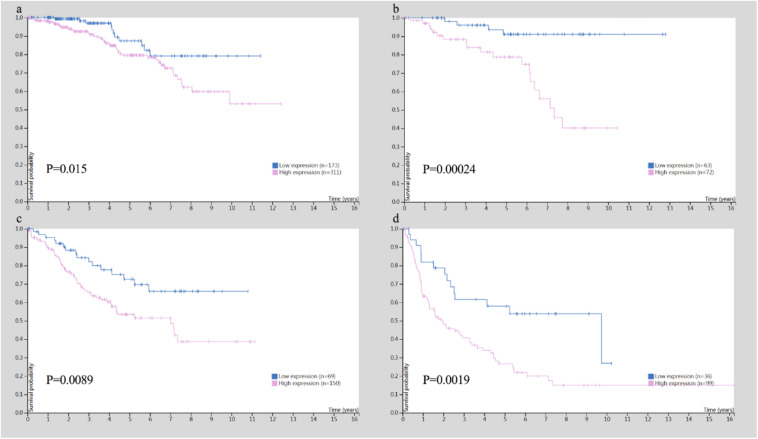
Survival analysis for TNM stage in colon and rectal cancer. **(A)** Survival analysis for MYC in stage I. **(B)** Survival analysis for MYC in stage II. **(C)** Survival analysis for MYC in stage III. **(D)** Survival analysis for MYC in stage IV.

**FIGURE 11:**
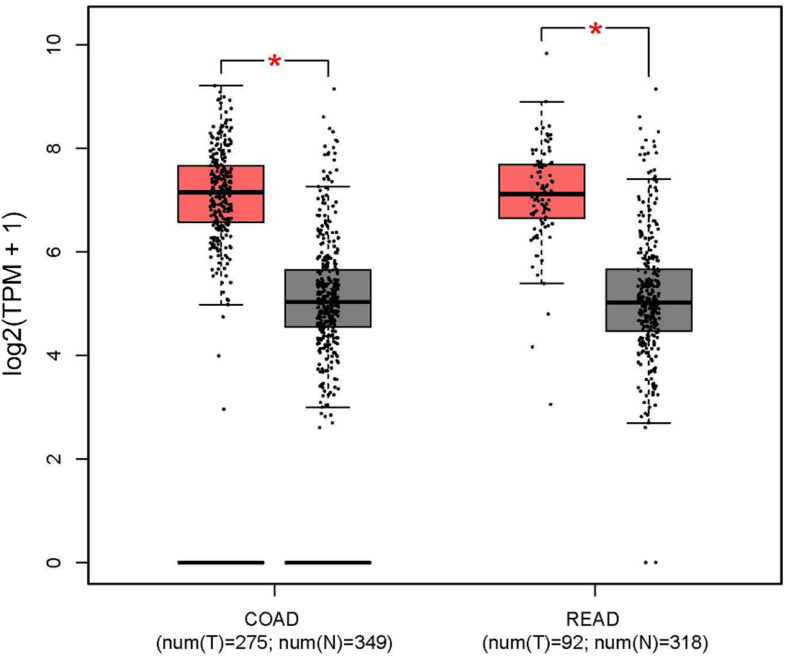
MYC expression level in colon and rectal cancer from the GEPIA database (red box: tumor; gray box: normal). COAD, colon adenocarcinoma; READ, rectum adenocarcinoma.

In addition, the Human Protein Atlas database was utilized to confirm the MYC prognostic value according to TNM stage (Figure 10). The data in the Human Protein Atlas database are mainly extracted from TCGA. The MYC expression was associated with a gradually decreased survival from stage I (5-year survival high 80%, 5-year survival low 87%), stage II (5-year survival high 79%, 5-year survival low 92%), stage III (5-year survival high 51%, 5-year survival low 69%) to stage IV (5-year survival high 19%, 5-year survival low 41%). The results showed that the MYC mRNA expression level was remarkably higher in CRC (Figure 11), so it is conceivable that a high MYC expression could serve as a risk factor for poor prognosis in CRC.

### Correlation Analysis Between MYC and Infiltrating Immune Cells

**FIGURE 12:**
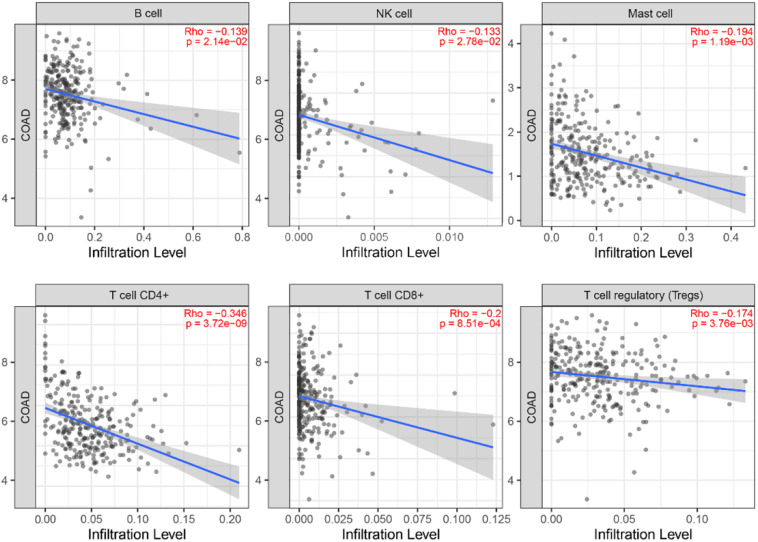
Correlation of MYC expression with immune infiltration level in the TIMER database.

In order to investigate the status of tumor-infiltrating lymphocytes, TIMER database was employed to explore whether the MYC expression was correlated with immune infiltration levels in CRC. The relationship between MYC expression and infiltrating immune cells is shown in Figure 12. The MYC expression levels showed a correlation with B, NK, Master, CD4+ T, CD8+ T, and regulatory T cell, and a high expression level of MYC was negatively correlated with CD8+ T cells.

### Screening of Tumor Suppressor Genes

Then, four candidate target genes in stage I, stage II, stage III, and stage IV were obtained by overlapping DNBs, down-regulated genes from stage I to IV, and human tumor suppressor genes (Table 7).

**TABLE 7:** Tumor suppressor genes in stage I to stage IV associated with colon and rectal cancer.

**Stage**	**Tumor suppressor genes**
Stage I	ZBTB16, MAL, LIFR, SLIT2
Stage II	ZBTB16, MAL, LIFR, SLIT2
Stage III	ZBTB16, MAL, LIFR, SLIT2
Stage IV	ZBTB16, MAL, LIFR, SLIT2

**FIGURE 13:**
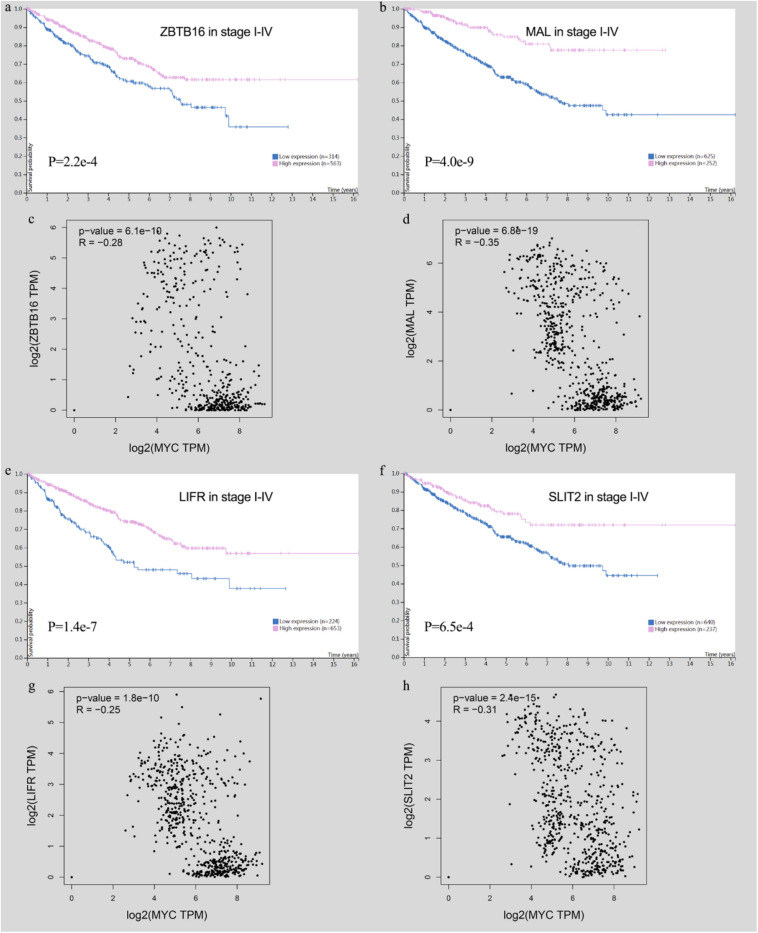
Survival analysis of tumor suppressor genes and the relationship with MYC). **(A)** ZBTB16 expression was correlated with high survival probability. **(B)** MAL expression was correlated with high survival probability. **(C)** ZBTB16 was negatively correlated with MYC. **(D)** MAL was negatively correlated with MYC. **(E)** LIFR expression was correlated with high survival probability. **(F)** SLIT2 expression was correlated with high survival probability. **(G)** LIFR was negatively correlated with MYC. **(H)** SLIT2 was negatively correlated with MYC.

High-expression tumor suppressor genes show a good prognosis in CRC, and they have a significant negative correlation with MYC in immune cells (Figure 13).

**FIGURE 14:**
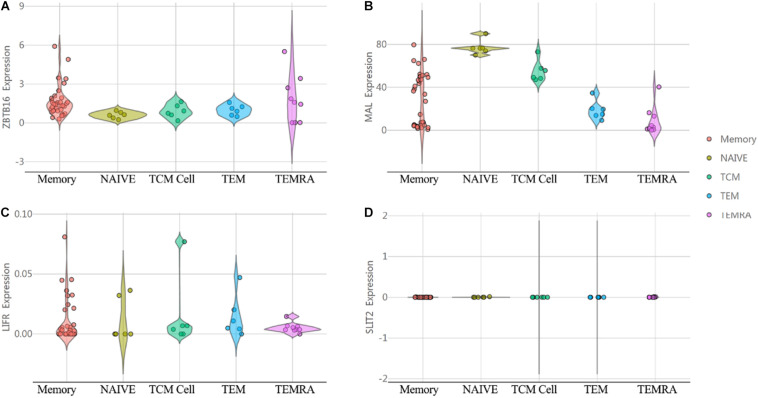
The expression of tumor suppressor genes in the human immune system. **(A)** ZBTB16. **(B)** MAL. **(C)** LIFR. **(D)** SLIT2.

**FIGURE 15:**
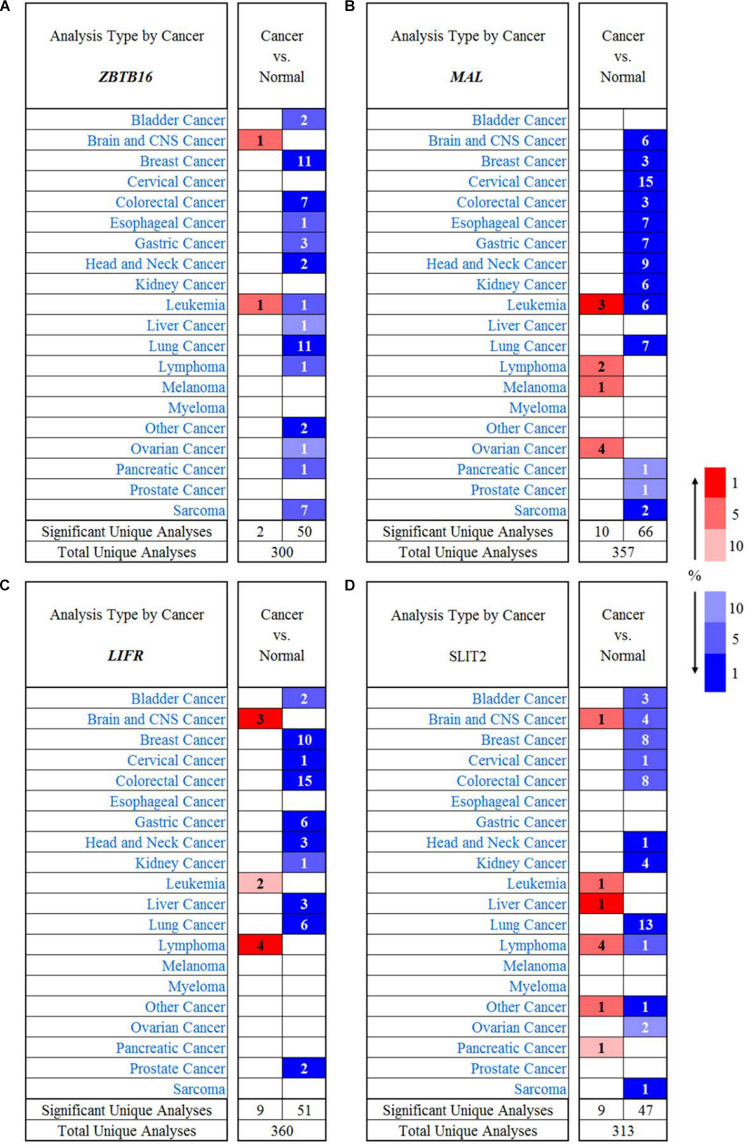
The expression of tumor suppressor genes in different types of human cancers. **(A)** ZBTB16 in different cancer data sets compared with normal tissues in the Oncomine database. **(B)** MAL in different cancer data sets compared with normal tissues in the Oncomine database. **(C)** LIFR in different cancer data sets compared with normal tissues in the Oncomine database. **(D)** SLIT2 in different cancer data sets compared with normal tissues in the Oncomine database.

In order to investigate the immunological mechanisms of tumor suppressor genes, the 10KIP (10,000 Immunomes Project) database (Zalocusky et al., 2018) was used to validate these tumor suppressor genes (Figure 14). Other than SLIT2 expression, ZBTB16, MAL, and LIFR expressions have a significant expression level with memory, naive, tcm, tem, and temra cell. Furthermore, the Oncomine database shows that the expression of tumor suppressor genes was significantly high in CRC (Figure 15).

## Discussion

Cancers are often initiated by some key genetic events, such as activated proto-oncogenes or inactive tumor suppressor genes (Casey et al., 2017), so elucidating the underlying molecular mechanism for these events is critically important for diagnosis and treatment in CRC. Although microarray and high-throughput sequencing has been widely used to predict the potential therapeutic targets for CRC, there is little research on identifying the biomarkers for the critical transition stage. In this study, we conducted a theoretical and *in silico* analysis of CRC development using the DNB algorithm. By applying the DNB algorithm in RStudio 1.1.453, we identified 98 DNBs that may herald the imminent critical transition in different tumor stages and provide new insights for the theory and practice studies in CRC. Moreover, the key member of the DNBs was calculated by PPI analysis and validated by public bioinformatics databases.

This study attempts to identify the DNBs for CRC. Dynamic network biomarker theory was performed in this study to recognize the key state of CRC from the time of stage I to stage IV. A joint bioinformatics analysis method was used for indicating tumor suppressor genes associated with DNBs in CRC; this may provide a set of useful targets for future investigation into the molecular mechanisms and biomarkers. Lastly, the critical DNBs and tumor suppressor genes in different tumor stages were detected and validated. The DNBs show that MYC might serve as a biomarker for the precise diagnosis and treatment of CRC in the future. The tumor suppressor genes were ZBTB16, MAL, LIFR, and SLIT2. These may contribute to the finding of molecular mechanisms underlying the initiation and development of CRC.

MYC proto-oncoproteins, including c-MYC, MYCN, and MYCL, perform their functions in cell proliferation, growth, death, angiogenesis, invasion, transformation, and metastasis (Wahlström and Arsenian Henriksson, 2015). MYC is a transcription factor that controls the expression of a large number of genes involved in cell proliferation, growth, angiogenesis, invasion, and metastasis (Walz et al., 2014). There is controversy about whether c-MYC can predict the prognosis of CRC (He et al., 2018). In all cancers, CRC seems to be particularly dependent on c-Myc activity. The transcription target c-Myc of the Wnt signaling pathway is up-regulated in up to 80% of CRC, which is mainly due to the loss of the Apc tumor suppressor (Myant and Sansom, 2011). Most coding mutations affect the stability of MYC proteins, thereby increasing their levels rather than altering the basic role of MYC (Elisha-Feil et al., 2006; Wierstra and Alves, 2008). It is reported that spermine synthase (SMS), in cooperation with MYC, can maintain CRC cell survival, so the combined inhibition of SMS and MYC signaling may be a promising strategy for inducing synergistic apoptosis and tumor regression (Guo et al., 2020).

MYC does not require changes to the protein sequence to cause cancer; it instead becomes an oncogene through abnormal expression (Love et al., 2012). MYC is a positive effector of tissue inflammation and can cause immune diseases (Williamson et al., 2006; Wang et al., 2011; Mckeown and Bradner, 2014; Gilhus et al., 2016). In T cells, it is involved in the proliferation, function, and lytic capacity of CD8 T lymphocytes (Ferrari et al., 1985; Grausz et al., 1986; Gamble et al., 1990; Selvatici et al., 1990; Nie et al., 2012). In activated primary T lymphocytes, MYC drives metabolic reprogramming through a MYC-dependent metabolic pathway that links glutamine breakdown to the biosynthesis of polyamines. However, MYC can also induce anti-inflammatory responses through macrophage function (Wang et al., 2011; Mckeown and Bradner, 2014). c-MYC has been shown to be expressed in humans as a macrophage classified as M2 (Pello et al., 2012) and plays a key role in macrophage polarization and tumor-associated macrophage function (Kim et al., 2015). Exposure to an anti-inflammatory response results in “alternatively activated” macrophage-M2 cells (Mosser and Edwards, 2008; Martinez et al., 2009), which is associated with anti-inflammatory properties, angiogenesis, and tissue remodeling (Kelly-Spratt et al., 2011). The c-MYC protein represents an early molecular marker of cell cycle activity, which may be important in the development of immune diseases (Trop-Steinberg and Azar, 2018). A study on immunohistochemical staining for metastatic colorectal cancer found that patients with a high expression level of c-MYC had a significantly lower progression-free survival time and overall survival than those with reduced c-MYC expression (Strippoli et al., 2020). In summary, these findings indicate that the uncertain role of MYC function warrants further investigation (Yunos et al., 2020).

ZBTB16 (zinc finger and BTB domain containing 16) is a member of the Krueppel C2H2 zinc finger protein family and encodes a zinc finger transcription factor that contains nine Kruppel zinc finger domains at the carboxy terminus. The protein is located in the nucleus, participates in the cell cycle process, and interacts with histone deacetylase. The specific situation of abnormal gene rearrangement at this locus is related to acute promyelocytic leukemia (The National Center for Biotechnology Information, 2020b). ZBTB16 acts as a transcriptional repressor (Lin et al., 2013). It plays a key role in the development and/or maintenance of myeloid maturity and other differentiated tissues. A potential substrate recognition component of the E3 ubiquitin protein ligase complex can mediate the ubiquitination of target proteins and subsequent proteasome degradation (Furukawa et al., 2003).

MAL (Mal, T cell differentiation protein) is a highly hydrophobic integral membrane protein that belongs to the MAL family of protein lipids. This protein is a candidate connexin in T cell signal transduction and has been located in the endoplasmic reticulum of T cells. In addition, the protein lipid is located in the tight myelin sheath of cells in the nervous system and is related to the biogenesis or function of the myelin sheath. The protein plays a role in the formation, stabilization, and maintenance of membrane microdomains rich in glycosphingolipids. The down-regulation of MAL is associated with a variety of human epithelial malignancies. Alternative splicing produces four transcript variants that differ from each other by the presence or the absence of alternately spliced exons 2 and 3 (The National Center for Biotechnology Information, 2020a).

LIFR (LIF receptor alpha) encodes proteins belonging to the type 1 cytokine receptor family. The protein combines with the high-affinity transformant subunit gp130 to form a receptor complex that mediates the action of a leukemia inhibitory factor, which is a multifunctional cytokine that participates in cell differentiation, proliferation, and, in adults and embryos, survival. Mutations in this gene cause Schwartz-Jampel syndrome type 2, which is a disease of curved bone dysplasia. Translocation involves the promoter of this gene. LIFR is a signaling molecule. The soluble form inhibits the biological activity of LIF by blocking the binding of LIF to the target cell receptor. Multiple splice variants encoding the same protein have been found for this gene (The National Center for Biotechnology Information, 2020a).

SLIT2 (slit-directed ligand 2) encodes a member of the slit family of secreted glycoproteins, which is a ligand of the Robo family of immunoglobulin receptors. Slot proteins play a highly conserved role in axon guidance and neuron migration and may also be functional in other cell migration processes, including leukocyte migration. Members of the SLIT family are characterized by an N-terminal signal peptide, four leucine-rich repeats, nine epidermal growth factor repeats, and a C-terminal cysteine junction. The proteolytic processing of this protein produces an N-terminal fragment containing four leucine-rich repeats, five epidermal growth factor repeats, and a C-terminal fragment containing four epidermal growth factor repeats and a cysteine junction. Both full-length and cleaved proteins are secreted extracellularly and can play a role in axonal rejection and other specific processes. Alternative splicing results in multiple transcript variants (The National Center for Biotechnology Information, 2020a). SLIT2 acts as a molecular guideline for cell migration during axonal navigation involving the ventral midline of the neural tube and axonal projection into different regions. SLIT2 appears to be necessary for midline guidance of the forebrain, and it acts as a repulsive signal to prevent the protruding axons of the olfactory bulb from improperly crossing the midline. In the development of the spinal cords, SLIT2 may play a key role in regulating the response to netrin as it guides the commissural axons to the floor. Only the commissural axons that cross the midline respond to SLIT2 *in vitro*.

However, there are several limitations that should be acknowledged in this study. The DNBs and the tumor suppressor genes were only validated in the TCGA database, so further molecular experiments are needed to better confirm the theoretical and *in silico* prediction. In conclusion, we hope that this study may provide some evidence for the future genomic treatment of CRC from new insights.

## Data Availability Statement

All datasets presented in this study are included in the article/Supplementary Material.

## Author Contributions

YT and SD conceived the study. YS collected the data. CX reviewed the algorithm. YT wrote the R code and the manuscript. All the authors read and approved the final manuscript.

## Conflict of Interest

The authors declare that the research was conducted in the absence of any commercial or financial relationships that could be construed as a potential conflict of interest.

## Abbreviations

CRC: colon and rectal cancer
GEO: Gene Expression Omnibus
DEG: differentially expressed genes
PPI: protein–protein interaction
COADREAD: TCGA colon and rectal cancer gene expression profile
COAD: colon adenocarcinoma
READ: rectum adenocarcinoma
DNB: dynamic network biomarker
WGCNA: weighted gene co-expression network analysis
DAVID: Database for Annotation Visualization and Integrated Discovery
GO: gene ontology
KEGG: Kyoto Encyclopedia of Genes and Genomes
STRING: Search Tool for the Retrieval of Interacting Genes
TCGA: The Cancer Genome Atlas
BP: biological process
BH: Benjamini and Hochberg
log2FC: log2-fold change
SD: standard deviation
PCC: Pearson coefficient
CI: composite index.
